# Socio-epidemiological and land cover risk factors for melioidosis in Kedah, Northern Malaysia

**DOI:** 10.1371/journal.pntd.0007243

**Published:** 2019-03-18

**Authors:** Muhammad Radzi Abu Hassan, Norasmidar Aziz, Noraini Ismail, Zainab Shafie, Benjamin Mayala, Rose E. Donohue, Subhada Prasad Pani, Edwin Michael

**Affiliations:** 1 Clinical Research Centre, Hospital Sultanah Bahiyah, Alor Setar, Malaysia; 2 Internal Medicine, Hospital Sultanah Bahiyah, Alor Setar, Malaysia; 3 Microbiology Unit, Hospital Sultanah Bahiyah, Alor Setar, Malaysia; 4 Demographic and Health Surveys (DHS) Program, ICF, Rockville, Maryland, United States of America; 5 Department of Biological Sciences, University of Notre Dame, Notre Dame, Indiana, United States of America; 6 Microbiology Department, Quest International University, Ipoh, Malaysia; University of Texas Medical Branch, UNITED STATES

## Abstract

**Background:**

Melioidosis, a fatal infectious disease caused by *Burkholderia pseudomallei*, is increasingly diagnosed in tropical regions. However, data on risk factors and the geographic epidemiology of the disease are still limited. Previous studies have also largely been based on the analysis of case series data. Here, we undertook a more definitive hospital-based matched case-control study coupled with spatial analysis to identify demographic, socioeconomic and landscape risk factors for bacteremic melioidosis in the Kedah region of northern Malaysia.

**Methodology/Principal findings:**

We obtained patient demographic and residential information and clinical presentation and medical history data from 254 confirmed melioidosis cases and 384 matched controls attending Hospital Sultanah Bahiyah (HSB), the main tertiary hospital of Alor Setar, the capital city of Kedah, during the period between 2005 and 2011. Crude and adjusted odds ratios employing conditional logistic regression analysis were used to assess if melioidosis in this region is related to risk factors connected with socio-demographics, various behavioural characteristics, and co-occurring diseases. Spatial clusters of cases were determined using a continuous Poisson model as deployed in SaTScan. A land cover map in conjunction with mapped case data was used to determine disease-land type associations using the Fisher’s exact test deploying simulated *p*-values. Crude and adjusted odds ratios indicate that melioidosis in this region is related to gender (males), race, occupation (farming) and co-occurring chronic diseases, particularly diabetes. Spatial analyses of disease incidence, however, showed that disease risk and geographic clustering of cases are related strongly to land cover types, with risk of disease increasing non-linearly with the degree of human modification of the natural ecosystem.

**Conclusions/Significance:**

These findings indicate that melioidosis represents a complex socio-ecological public health problem in Kedah, and that its control requires an understanding and modification of the coupled human and natural variables that govern disease transmission in endemic communities.

## Author summary

Although the public health significance of melioidosis as a particularly highly fatal emerging infectious disease threat in the tropics is being recognized, data on the risk factors and the geographic distribution of the disease is still limited. Previous studies have also largely been based on the investigation of case series data. Here, we extend these studies by employing a hospital-based matched case-control study to carry out a more robust examination of the demographic, socioeconomic, and landscape risk factors that may govern melioidosis incidence in the Alor Setar area of Kedah, State, a key disease endemic region of Malaysia. Our results indicate that melioidosis in northern Malaysia is significantly associated with gender, race, occupation, co-occurring chronic disease as well as living in risky landscapes, with disease risk increasing and declining non-linearly with the degree of ecosystem modification. These findings denote that the disease represents a complex socio-ecological public health problem in Kedah, and that its control requires an understanding and modification of both the human and natural variables that underlie disease transmission in this setting.

## Introduction

Melioidosis, once thought to be restricted to Southeast Asia and northern Australia [1], is now increasingly diagnosed in other tropical regions, including across Africa, the Caribbean, and other parts of Asia [2, 3]. Thriving in soil and surface water, the causative *Burkholderia pseudomallei* saprophytic bacterium can bear extreme environments, including enduring starvation for long durations, a likely biological factor underlying its survival and potential transmission across broad geographical regions [4]. The main mode of transmission involves physical contact of lesions with contaminated reservoirs; however, inhalation or ingestion of particles in the air can also serve as dissemination mechanisms. *B*. *pseudomallei* has been found to be the causal agent for approximately 20% of community acquired bacteremias in north- eastern Thailand, [4] but the pathogen can also induce a wide spectrum of clinical manifestations ranging from pneumonia, internal organ abscesses to septicemia. The disease is notably lethal, with overall mortality rates found to range anywhere between 19 and 54 percent in different communities [2, 5].

Previous work has shown that peninsular Malaysia may be at high risk for the disease, with hospital cases recorded from practically all regions of the country [6–11], and calculated annual incidences ranging from 4.3 per 100,000 in the eastern state of Pahang [6] to as high as 16.35 per 100,000 recently reported by us in the northwestern agricultural state of Kedah [7]. These studies have also highlighted the high fatality rate, as well as provided data suggestive of the myriad social and ecological factors that may govern disease transmission in this region [6–11].

Despite the extent and potentially high public health significance of the disease in the country, definitive information on risk factors are still constrained by the fact that previous studies have invariably focused on analyses of patient case records. Although such case series-based studies are useful for estimating relative disease incidences and for quantifying the prevalence of exposure or risk factors associated with the disease [12], these analyses are limited by the quality of patient selection, observation period, and time-invariant or fixed confounder effects [12, 13].

Here, we extend our previous case series study by employing a hospital-based matched case-control investigation to carry out a more powerful and definitive examination of the demographic, socioeconomic, and landscape risk factors that may govern melioidosis incidence in the Alor Setar region of Kedah state. Although it is well-known that melioidosis in Southeast Asia is associated with rice farming [2, 14], the relative risk for disease across the major landscapes occurring in this region, including in the case of Kedah, has never been quantified. We therefore analyzed the spatial distribution of patient-cases in this study to both identify for the first time the areas of high risk in the state [15], and to assess the landscape features that are likely to be associated with this soil and water-mediated infectious disease in this region.

## Methods

This was a retrospective matched case-control study conducted using melioidosis cases and controls attending Hospital Sultanah Bahiyah (HSB), the main tertiary hospital of Alor Setar, the capital city of Kedah, during the period between 2005 and 2011. Melioidosis is a notifiable disease in Kedah with HSB serving as the primary reference hospital for the state. We obtained patient demographic and residential information, clinical presentation, and medical history for all selected cases and controls from the relevant HSB patient registries.

### Selection of case-patients and controls

All case-patients were extracted from the Melioidosis Registry, established in 2005 and containing all confirmed melioidosis cases referred to HSB. Confirmation of melioidosis at HSB is rigorous and is done by culture, serology, or a combination of both tests [7]. Microbial detection of *B*. *pseudomallei* in blood cultures is achieved using the BACTEC9420 Instrumented Blood Culture System (Fluorescent Series, Becton Dickinson). Cultures of other bodily fluids were performed using blood agar and MacConkey’s medium, and the API 20 NE biochemical identification system (BioMérieux) for *B*. *pseudomallei*. Serology tests are based on detection of *B*. *pseudomallei* using the Indirect Fluorescent Antibody (IFA) method. Since culture tests are the gold standard for diagnosis, only culture positive cases were included in this study, resulting in an initial selection of 254 out of the original 488 cases from the Melioidosis Registry for the above study period. By contrast, 384 controls were initially collected from patients admitted to the orthopedics department in HSB at the same time as the selected case-patients. These controls were matched with case-patients for age, gender, race, home address, and admission date to HSB (+/- one week). Addresses were matched to the nearest village or street. Following the matching effort, we obtained 242 matched pairs of cases and controls.

### Statistical analysis of socio-epidemiological data

Demographics, risk factors, including smoking, alcohol usage and occupation, and underlying illnesses, were compared between controls and case-patients. Occupation was categorized into three classes representing low to high occupational risk for contracting melioidosis based on likely exposure. Those considered to have low occupational risk were in sales, executive positions, academia, and other job roles with minimal outdoor exposure; the medium group consisted of children, housewives, and those with service jobs; while those in the high occupational risk group were solely farmers. For continuous variables, either the Student’s t test or the Mann-Whitney U test was used, and for categorical variables either the Pearson χ2 test or Fisher’s exact tests was used as required. A conditional logistic regression was used to calculate the adjusted odds ratios (OR) for associations of melioidosis with different underlying illnesses. All analyses were performed using SPSS 20 and R 3.0.1 [16, 17].

### Spatial analysis

Cases were mapped on Google Earth based on patient address to obtain latitude and longitudinal coordinates. Overall, locations were identified for 175 case-patients; controls were not mapped because of locational matching with case-patients. The original latitude and longitudinal coordinates were then projected to Kertau UTM Zone 47 for Malaysia to create a case distribution map using ArcGIS 10.1 [18]. The presence of significant clusters in the spatial case data was assessed using the scan statistics feature in SaTScan 9.0 [19]. A continuous Poisson model, which tests the hypothesis that cases follow a homogeneous spatial Poisson process with constant density throughout the study area [20], was applied with the following parameters: scanning for areas with high rates in a circular manner, a maximum spatial cluster size set to 50% of the population at risk, and no geographical overlap [17].

In order to determine the prevalence of melioidosis within different landscapes occurring in the study area, we first overlaid the case point data onto a 5 arc minutes land cover map of East Asia and the Pacific [21], clipped to cover the Kedah state boundary. This allowed the count of cases falling in each land cover type. A gridded population map of Kedah was then extracted from the Gridded Population of the World [22] and overlaid on the land cover map in order to derive the population count within each land cover type. The melioidosis prevalence in each land cover classification was estimated using the following equation: (number of cases/population count) * 100,000. Spatial variation in the prevalence of the disease between the different land cover types was assessed by applying the Fisher’s exact test with simulated *p*-values based on 2000 replicates [14].

The proportions of each land cover type within and outside the identified disease cluster were calculated using ArcGIS [18], and statistical differences in the composition of proportionate landcover types within and outside the cluster were evaluated using a Dirichlet regression model for testing variations in compositional data between categorical variables [23].

### Ethics statement

The project was approved by the National Institute of Health (NIH) and Malaysian Research and Ethics Committee (MREC). All patient data used in this study was anonymized.

## Results

### Case-patients and controls

Demographic characteristics, and underlying risk factors and illnesses, are displayed in Table 1 for the matched cases and controls. Overall, there was a preponderance of males (76.4%), the predominant race was Malay (93.0%), and the mean age was 46.6, with males slightly older than females (Table 1). Subject occupation appeared to play a significant role in disease acquisition, with case-patients found to be engaged proportionately more in the high risk occupation of farming (16.9% in case-patients versus 10.3% in controls: χ2 = 4.49, *p* < 0.034), while they were significantly underrepresented in the low risk occupations of sales, executive positions and academics (10.7% versus 22.3% in controls: χ2 = 11.74, *p* < 0.001).

**Table 1:** Characteristics of matched cases and controls including demographic characteristics, risk factors, underlying illnesses, and mortality.

	Melioidosis Cases (no. = 242) (%^§^)	Melioidosis Controls (no. = 242) (%^§^)	*p*-value	x^2^
**Mean Age**				
Male ^‡^	47.75 (16.6)	47.82 (16.4)		
Female ^‡^	42.95 (18.6)	42.93 (18.7)		
**Male**	185 (76.4)	185 (76.4)		
Malay	170 (70.2)	170 (70.2)		
Chinese	9 (3.7)	9 (3.7)		
Indian	3 (1.2)	3 (1.2)		
Others	3 (1.2)	3 (1.2)		
**Female**	57 (23.6)	57 (23.6)		
Malay	55 (22.7)	55 (22.7)		
Chinese	0	0		
Indian	1 (0.4)	1 (0.4)		
Others	1 (0.4)	1 (0.4)		
**Occupation**				
Low**	26 (10.7)	54 (22.3)	<0.001	11.74
Medium	69 (28.5)	75 (31.0)	0.60	0.36
High*	41 (16.9)	25 (10.3)	0.04	4.49
Unknown	106 (43.8)	88 (36.4)	0.12	2.79
**Smoker**				
Yes	52 (21.5)	46 (19.0)	0.678	0.17
No	109 (45.0)	87 (36.0)		
Unknown	81 (33.5)	109 (45.0)		
**Alcohol Consumption**				
Yes	3 (1.2)	2 (0.8)	0.202	0.65
No	239 (98.8)	240 (99.2)		
**Underlying Illnesses**				
Diabetes Mellitus**	145 (59.9)	73 (30.2)	<0.001	43.27
Chronic Renal Failure**	19 (7.9)	5 (2.1)	0.003	8.59
Chronic Lung Disease	3 (1.2)	1 (0.3)	0.315	1.01
HIV/AIDs	4 (1.7)	1 (0.3)	0.177	1.82
Immunocompromised States	4 (1.7)	0		
Other Diseases	87 (36.0)	79 (32.7)	0.045	4.03

Data shown as no. (%); p-value <0.05 * and <0.01 **

^‡^ data shown as mean (standard deviation)

^**§**^ percentages calculated based on total subjects (no.) for each disease group.

A few of the conditions included in the “Other Diseases” category are hypertension, hypercholesterolaemia, heart disease, hepatitis B and C, thalassemia, and tuberculosis. A majority of the deceased patients presented with various combinations of the underlying illnesses, most commonly, diabetes mellitus and chronic renal failure, as well as, diabetes mellitus and other diseases.

While no significant associations between case-patients and either alcohol use or smoking were found for this population, varying degrees of statistically significant relationships, by contrast, were found between case-patients and diabetes (χ2 = 43.269, *p* < 0.001), chronic renal failure (χ2 = 8.593, *p* = 0.003), and other immunocompromised states (χ2 = 4.03, *p* = 0.045). Crude ORs (Table 2) from running ordinary univariate logistic regressions supported these findings for diabetes, (OR = 3.46, 95% CL:2.38–5.04) and chronic renal failure (OR = 4.04, 95% CL:1.30–2.05), but not other diseases (chronic lung failure, HIV and other immunocompromised states) (OR = 1.20, 95% CL:0.83–1.75). By contrast, adjusted ORs from the conditional logistic model showed that all three (diabetes: OR = 4.13, 95% CL:2.62–6.51; chronic renal failure: OR = 1.95, 95%CI 1.19–3.19; and other diseases: OR = 10.0, 95% CL:1.28–78.12) comprised significant risk factors for the disease. They also show that the odds of acquiring melioidosis is likely to be 4 times higher among diabetics and about 2 times higher among patients with chronic renal failure.

**Table 2:** Crude OR and adjusted OR from conditional logistic regression for matched cases and controls.

	Crude O.R	95% CI	Adjusted O.R	95% CI	*p*-value
Diabetes	3.46	2.38–5.04	4.13	2.62–6.51	0.00
Chronic Renal Failure	4.04	1.30–2.05	1.95	1.19–3.19	0.01
Other ^‡^	1.20	0.83–1.75	10.00	1.28–78.12	0.03

^‡^ includes chronic lung failure, HIV, and immunocompromised states

### Case-fatality

The case-fatality ratio among culture-confirmed melioidosis cases was 41.8% (95% CL: 35.5–48.4) while the case-fatality ratio among melioidosis-suspected cases was 19.7% (95% CL: 14.7–25.9) (Table 3). Risk factors which significantly affected the relative risk of death among the confirmed cases include age group (with risk of death significantly low amongst the youngest section of the present cases), having diabetes mellitus, having an unknown-risk for occupation, or having an unknown smoking status. Among melioidosis-suspected cases, the risk factors significantly affecting the risk of dying include having an underlying infection of diabetes mellitus or chronic renal failure.

**Table 3:** Case-fatality ratio and relative risk of death by socio-demographic and clinical factors among melioidosis culture-confirmed and suspected cases.

	Melioidosis Confirmed Cases (n = 220)				Melioidosis Suspected Cases (n = 193)			
	n^§^	Deaths	RR	95% CI	n^§^	Deaths	RR	95% CI
**Case Fatality Ratio**^‡^	220	92	41.8	35.5–48.4	193	38	19.7	14.7–25.9
**Sex**								
Male	163	68	Ref.	—	124	25	Ref.	—
Female	57	24	1.01	0.71–1.44	69	13	0.93	0.51–1.71
**Age**								
0–24	34	9	0.53	0.29–0.97*	26	2	0.32	0.08–1.26
25–54	118	49	0.83	0.60–1.14	85	16	0.77	0.43–1.38
55+	68	34	Ref.	—	82	20	Ref.	—
**Race**								
Malay	199	85	Ref.	—	175	33	Ref.	—
Chinese	9	3	0.78	0.31–1.99	11	3	1.45	0.53–3.98
Indian	7	3	1.00	0.42–2.40	3	0	0.00	NA
Others	5	1	0.47	0.08–2.72	4	2	2.65	0.95–7.41
**Occupation**								
Low	24	6	Ref.	—	32	5	Ref.	—
Medium	68	19	1.12	0.51–2.47	72	12	1.07	0.41–2.78
High	34	12	1.41	0.62–3.23	28	5	1.14	0.37–3.54
Unknown	94	55	2.34	1.15–4.78*	61	16	1.68	0.68–4.16
**Smoker**								
Yes	41	16	1.13	0.71–1.79	40	11	1.56	0.82–2.99
No	107	37	Ref.	—	108	19	Ref.	—
Unknown	72	39	1.57	1.12–2.19*	45	8	1.01	0.48–2.14
**Underlying Illnesses**								
Diabetes Mellitus	128	61	1.41	1.01–1.99*	88	23	1.83	1.02–3.29*
Chronic Renal Failure	18	10	1.37	0.88–2.14	10	5	2.77	1.39–5.54*
Chronic Lung Disease	3	2	1.61	0.71–3.63	3	0	0	NA
HIV/AIDs	3	1	0.79	0.16–3.97	3	1	1.71	0.34–8.70
Immunocompromised States	5	2	0.96	0.32–2.83	2	0	0	NA
Other Diseases	83	30	0.80	0.57–1.12	58	14	1.36	0.76–2.43

^**§**^ Individuals who were transferred to a different hospital or discharged from the hospital at their own risk were excluded from the analysis as we do not know if they final clinical outcome was death

^‡^ data shown as case-fatality ratio rather than relative risk ratio

* indicates a p-value <0.05.

### Spatial analysis

**Fig 1:**
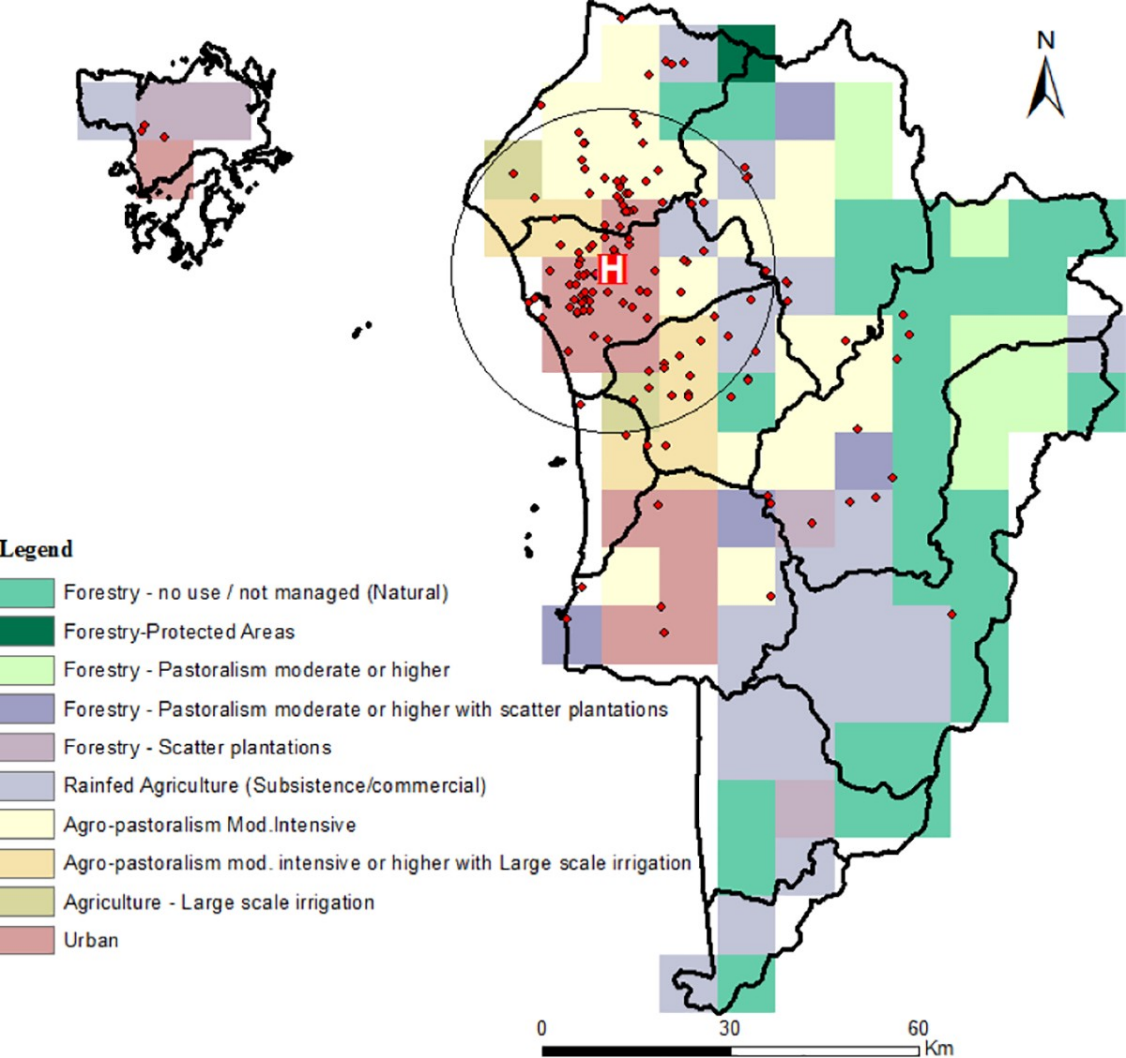
Population map of Kedah, Malaysia showing spatial distribution of melioidosis cases. Red block with “H” represents Hospital Sultanah Bahiya (HSB). Spatial scan statistic revealed the most likely disease cluster, black triangle (radius 0.23, *p*<0.001). No significant secondary clusters were discovered. Created with ArcGIS 10.1 [18] using district boundaries provided by the Department of Statistics, Malaysia. Land cover and population map layers were generated as described in Methods.

The spatial distribution of melioidosis cases superimposed on the Kedah land cover map is shown in Fig 1, and indicates a concentration of cases in the urban and semi-urban settings surrounding Alor Setar. The continuous Poisson spatial scan statistic detected one likely primary cluster (log likelihood ratio = 505.18, radius = 25.57 km, centroid coordinates = 100.467, 6.15, p<0.001) that included HSB (Fig 1). No significant secondary cluster was detected.

Table 4 shows the prevalence of the disease across the five major land types or landscapes expected to influence melioidosis transmission within the present study location. These land cover types were derived by combining the finer scale environments shown in Fig 1 (see details of the land cover combinations carried out as shown in Table 4), and might be considered to represent a gradient in the underlying environmental exposure risk to acquiring melioidosis. They also represent a graded increase in the degree of human modification of the natural ecosystem from forested areas to the urban setting. Table 4 shows that, as expected, melioidosis prevalence varied markedly between these land cover types, with results from the Fisher’s exact test showing this variation to be highly statistically significant (χ2 = 45.019, p< 0.0005).

**Table 4:** Prevalence of melioidosis within various land use types.

Land Use Type	Prevalence Rate (per 100,000)
Forestry	3.30
Mixed forestry and pastoralism	6.82
Mixed agriculture and pastoralism	16.17
Agriculture–large scale irrigation	21.04
Urban Areas^§^	9.93

^**§**^7 cases were removed from the urban prevalence estimation due to their participation in a high-risk occupation. (Fisher’s exact test showed significance with a simulated p-value of 0.0005)

**Fig 2:**
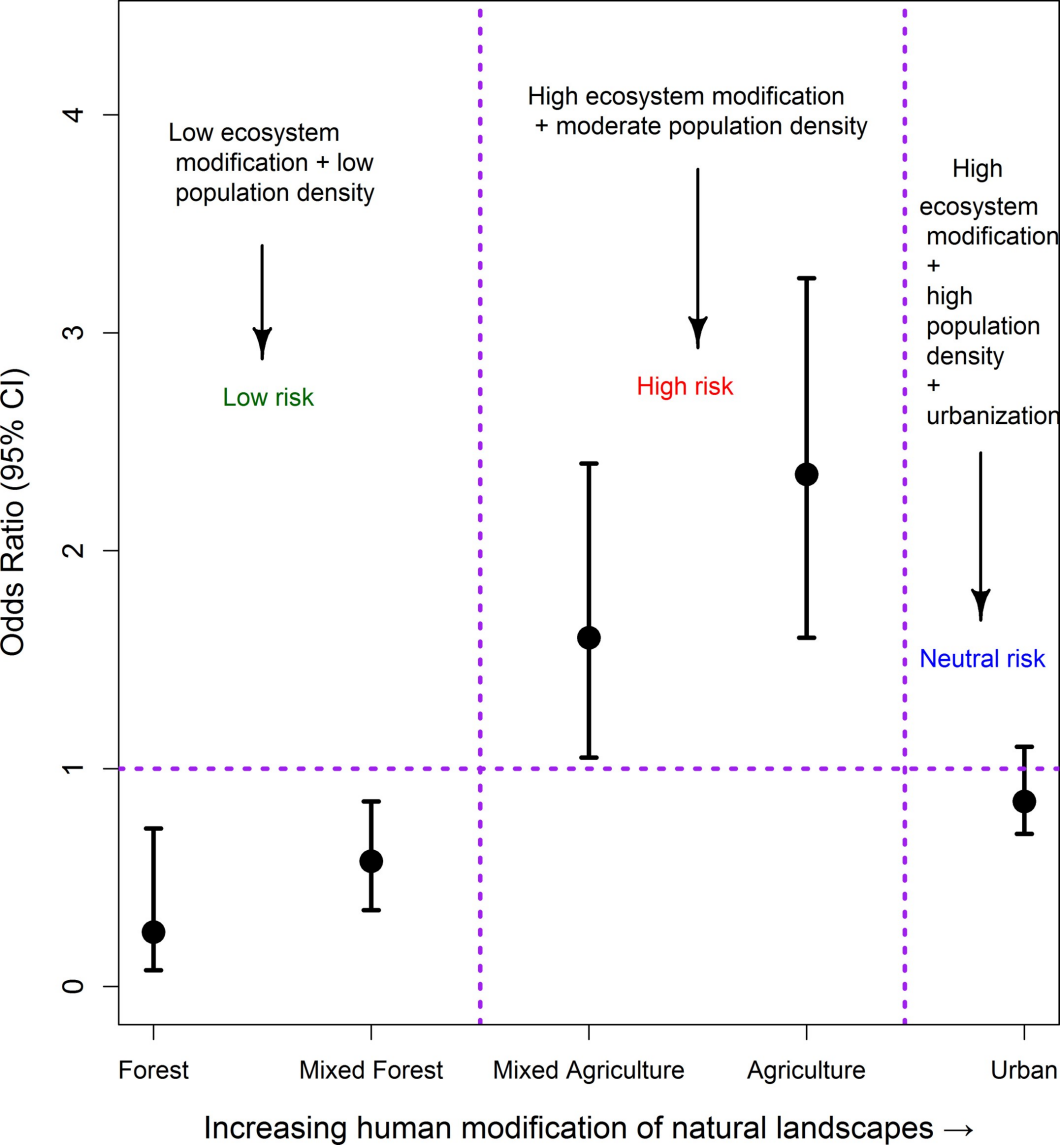
Increasing risk of melioidosis with escalating levels of human modification to the ecosystem of Kedah, Malaysia.

We estimated the risk of living in these environments by calculating an exposure OR based on the joint distributions of cases and populations at risk in each land type. This showed that while living in areas with large-scale irrigation-based agriculture represented the most risk for acquiring melioidosis (OR = 2.24, 95% CL:1.57–3.17), followed by mixed agriculture/pastoral environments (OR: 1.55, 95% CL:1.02–2.31); living in forest-associated areas, either the mixed forested (OR: 0.55, 95% CL:0.34–0.86) or forestry areas (OR: 0.28, 95% CL:0.08–0.73), protected against the disease. Living in urban Alor Setar also provided some protection, but this protection did not attain statistical significance (OR: 0.83, 95% CL: 0.59–1.13). Fig 2 plots the ORs in relation to the five land cover types, and illustrates the non-linearity in the impact of the investigated land cover types on melioidosis risk.

**Fig 3:**
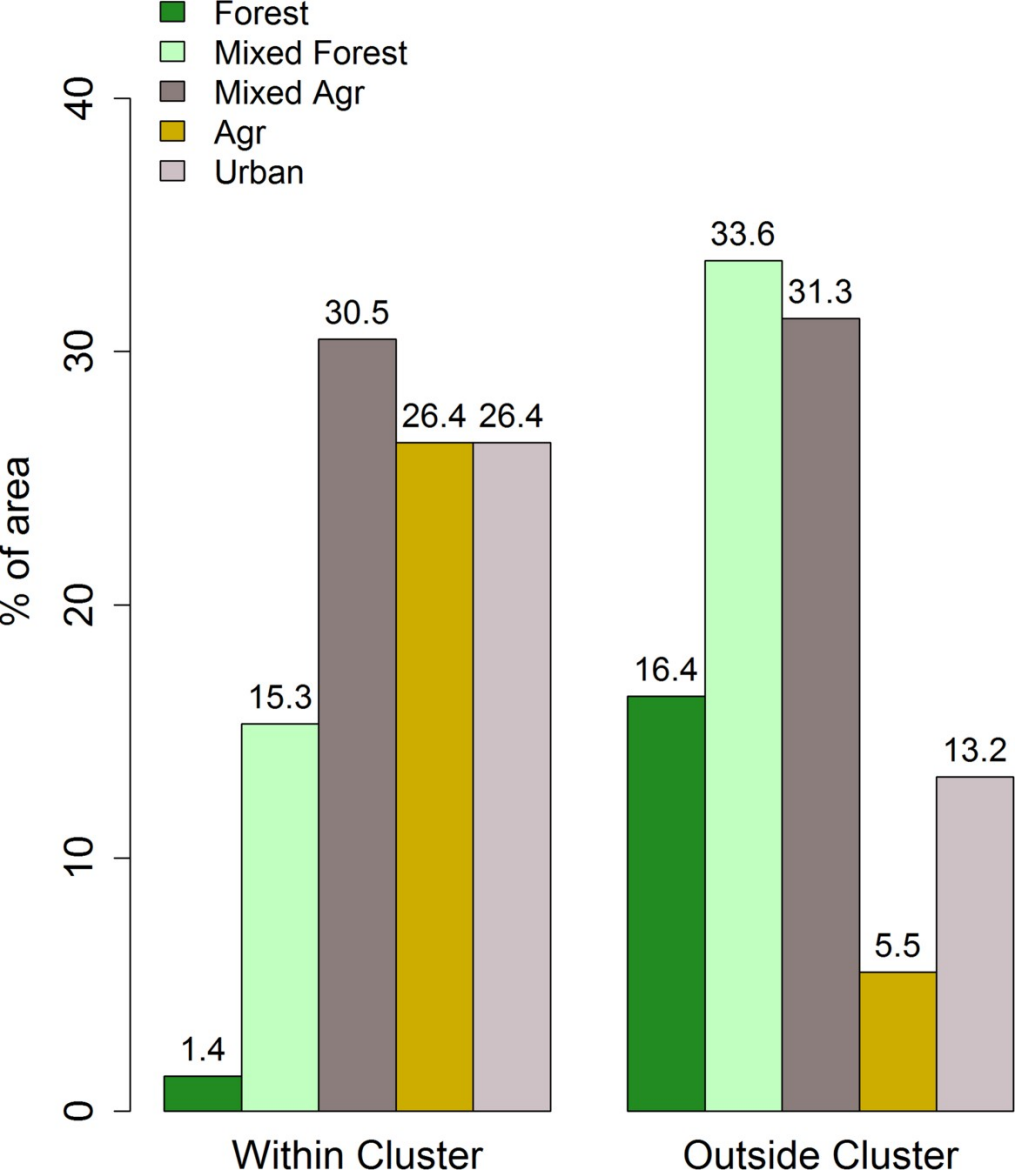
Percentage of land use types inside and outside of the primary cluster of melioidosis cases in Kedah, Malaysia.

Fig 3 shows the proportions of the five landscapes observed within the primary high infection cluster (Fig 1) in comparison to the proportions recorded outside the cluster. The proportions outside the cluster were obtained by calculating the respective areas of each land cover type occurring within a convex hull placed around the locations of cases outside the primary cluster (Fig 1). The results show that the proportions of each land cover type varied markedly within and outside the primary cluster, with high risk land cover types (mixed agriculture and in particular, agricultural areas) occurring proportionately more within the primary cluster (Dirichlet Regression with intercept only versus one including cluster effects: deviance = 220.5142, df = 5, p<0.001). A multivariate logistic regression analysis, controlling for the effects of age, gender and occupational differences between cases located within and outside the primary cluster, confirmed that the observed spatial clustering of melioidosis cases found in this study was a direct function of living in such “pathogenic” environments (Table 5).

**Table 5:** Adjusted OR from binary logistic regression comparing cases inside and outside of the primary cluster.

	O.R.	95% CI	*p*-value
Occupation (low)	1.48	0.56–3.88	0.43
Occupation (medium)	1.77	0.49–6.35	0.39
Occupation (high)	1.69	0.53–5.36	0.38
Diabetes	0.79	0.37–1.68	0.54
Land use	3.01	1.48–6.13	0.002
Age	1.01	0.99–1.03	0.54
Gender	0.98	3.96–2.40	0.96

## Discussion

Although Malaysia is increasingly recognized as an important endemic focus for melioidosis [7, 24], and previous investigations have highlighted the basic epidemiological features of the disease among cases, this is the first case-control study from that country conducted specifically to elucidate the socio-epidemiological and landscape risk factors, as well as magnitudes of their association, for bacteremic disease. In order to more effectively evaluate the impacts of these diverse etiological factors, we have also used an efficient matched case control study design to undertake the present analysis.

Our study populations were representative of the ethnic make-up of Kedah state, with 93.0 percent of study subjects being Malay. The Census of Malaysia reports an equal proportion of males and females within the state of Kedah [25]. However, 76.4 percent of the case-patients in our study population were males while 23.6 percent were females, supporting suggestions that there is a strong tendency for infections to occur in males in this region [24, 26]. The mean age of male subjects was slightly higher than that of females (Table 1), but since our previous study indicated that age may have a non-linear rise and fall relationship with cases [7], this age difference is unlikely to underlie the higher occurrence of the disease in males. On the other hand, as males in the present community engage more in activities related to greater contact with soil (e.g. farming, small-scale foodstuff or recreational gardening), this result corroborates previous conclusions [27, 28] that the elevated disease prevalence/incidence observed for males is due to their higher occupational exposure to the disease [27–29].

The strong occupationally-related hazard for acquiring melioidosis was further corroborated by our analysis of socio-economic risk factors for the disease (Table 1). Thus, while individuals engaged in activities with a generally low outdoor exposure appeared to be protected from the disease, those in occupations with higher outdoor exposure were at elevated risk of being infected and sick. In particular, the results indicate that due to a potentially prolonged exposure to soil sources and thus higher probability of inhalation, ingestion or inoculation of *B*. *pseudomallei*, rice farmers, a large proportion of who were also males, in this region form the most vulnerable group for infection. This finding is in line with results from other studies conducted in Southeast Asia with a comparable extensive rice farming agricultural profile as Kedah state [30, 31]. However, as our spatial analysis of landscape risk indicates, this observed association with occupation may be a complicated one. The key result here is that after controlling for occupation, individuals living in high risk environments, viz. agriculture/mixed agriculture regions, are still at higher risk for acquiring infection, suggesting that rather than occupation *per se* it is the place in which these activities take place that is more important.

Excess alcohol consumption has been identified as a predisposing factor for melioidosis in tropical Australian populations [26]. However, the religion most commonly practiced in Kedah is Islam, which prohibits practicing Muslims from the consumption of alcohol. Thus, only a diminutive one percent of our study group consumed alcohol, showing its insignificance as a risk factor in this particular population, as was also found to be the case in previous studies from Malaysia [10] and Singapore [32]. By contrast, our results reinforce the results from different communities in tropical Australia and across the Southeast Asia region, which indicate that patients with undermined immune systems, specifically those with diabetes and chronic renal diseases, are significantly at higher risk for developing melioidosis [4, 24–26]. The finding that diabetes is a risk factor for melioidosis is of particular concern in Malaysia due to its high diabetes prevalence and the observed trend of increasing prevalence [33], which has been attributed to the adoption of more “Westernized” diets and population shifts in exercise [34]. However, while patients with chronic lung disease were also at a significantly higher risk for the disease in the northern tropical Australian study setting, this subgroup was not a major constituent in our study (Table 1), and was also omitted in the Thailand investigation due to difficulties in risk estimation using retrospective records [25, 26]. These disparate results, and the finding from a concluded randomized control trial [35], has cast doubt on the involvement of the complex polymorphonuclear leukocyte (PMNL) network in increased risk to melioidosis observed amongst those with the above underlying diseases [29, 36–38], suggesting that research to uncover more complex mechanisms in diverse endemic populations must continue to be a priority if more effective therapeutic or other interventions are to be developed for controlling such deadly co-morbidities.

The overall case-fatality rates among culture-confirmed and suspected cases were 41.8% and 19.7%, respectively (Table 3), illustrating the lethal nature of the disease in Kedah communities despite the use of recommended standard antibacterial agents for therapy. For both confirmed and suspected cases, case-fatality rates did not differ significantly by sex or ethnicity but were higher for patients with diabetes and for suspected cases, patients with chronic renal failure (Table 3). These findings are similar to results from other studies carried out in Southeast Asia and Australia [26, 27].

Our analysis of the spatial distribution of melioidosis cases, indicated, firstly, the presence of a significant primary cluster of elevated cases occurring in close proximity to the main city of Alor Setar, where HSB is also located (Fig 1). While this may reflect the confounding effect of patient proximity to the hospital, the fact that HSB serves as the primary reference hospital for the disease in the state of Kedah suggests that this influence is unlikely to be the major cause of the identified disease cluster. This conclusion is further supported by the results of our analysis of the spatial risks associated with acquiring melioidosis for populations residing in different landscapes. The major finding here is that while human-unaltered forested areas are protective, the highest risks occurred with agricultural-related modifications of the environment, particularly in the case of large-scale irrigation-based agriculture, which in the Kedah context is primarily related to vast rice cultivation. Intriguingly, the results show that the risk of acquiring the disease declined, and indeed was neutral in the urban setting of Alor Setar, which may be considered to represent the most human modified of the land types studied here. These findings support the growing evidence from landscape-based epidemiological studies, which suggest that forest modification and fragmentation may be a major driver for the emergence of many vector- and water-borne diseases [39, 40]. We suggest that for melioidosis, the specific mechanisms, apart from variations in human population density, which could underlie this association may be primarily related to an increase in the habitat and hence abundance of *B*. *pseudomallei* as forest ecosystems are altered to produce agricultural land types. For example, the protection afforded by intact forests may be due to their ecohydrological functions that may regulate water- or soil-borne pathogen emergence by filtering pathogen-laden runoff and modulation of the amplitude of flows during seasonal rain [35]. However, we did not measure the presence or abundance of *B*. *pseudomallei* in the different land use types, so this additional analysis would need to be conducted to test our proposed hypothesis.

However, our finding that the risk of acquiring melioidosis may be reduced in the urban setting indicates that the association between infectious disease emergence and human modification of the natural ecosystem is complex and will depend on the specific pathogen. For melioidosis, we suggest that urban areas may represent a neutral risk setting because although on the one hand, they represent centers of high population density, this is countered by the presence of lower pathogen habitats, higher socio-economic development of the population, and better provision of health and other services.

This study has several limitations. Firstly, melioidosis may remain latent for years before symptoms develop; thus, the place of residence at the time of infection may differ from the place of current residence. Secondly, we selected controls from the orthopedics department, which may introduce a selection bias as individuals in the orthopedics department may not be representative of the entire melioidosis-negative population. Individuals in the orthopedics department were not clinically tested for the presence of *B*. *pseudomallei*; however, it is unlikely the controls are infected as melioidosis is a notifiable disease although they may be latently infected. An additional source of selection bias may have been introduced by the decision to recruit individuals from a single hospital; distance to hospital may play an important role in determining which individuals choose to seek treatment. However, melioidosis is a notifiable disease and clinically positive individuals are referred to HSB from all over Kedah state, so this may diminish the importance of the distance to hospital variable.

Overall, our results indicate that complex and interacting socio-ecological factors may underlie the transmission of melioidosis in Kedah. This conclusion also indicates that if we are to gain a fuller understanding of the components and their modification required to prevent the future emergence as well as reduction of the disease amongst at-risk populations in Malaysia, and elsewhere in the tropics, it is essential that we deploy an investigatory approach that examines melioidosis as an outcome of coupled human and natural variables acting at various spatial scales from the individual host to the environment.

## Supporting information

S1 Checklist STROBE checklist. (DOC)

## References

[1] Control CfD, Prevention. CDC Health Information for International Travel 2012: The Yellow Book: Oxford University Press; 2012.

[2] Cheng AC, Currie BJ. Melioidosis: Epidemiology, Pathophysiology, and Management. Clin Microbiol Rev. 2005;18(2):383–416. 10.1128/CMR.18.2.383-416.2005 15831829 PMC1082802

[3] Lo TJ, Ang LW, James L, Goh KT. Melioidosis in a Tropical City State, Singapore. Emerging Infect Dis. 2009;15(10):1645–7. 10.3201/eid1510.090246 PMC2866399. 19861063 PMC2866399

[4] Chaowagul W, White NJ, Dance DAB, Wattanagoon Y, Naigowit P, Davis TME, et al Melioidosis: A Major Cause of Community-Acquired Septicemia in Northeastern Thailand. J Infect Dis. 1989;159(5):890–9. 2708842 10.1093/infdis/159.5.890

[5] Peacock SJ, Limmathurotsakul D, Lubell Y, Koh GC, White LJ, Day NP, et al Melioidosis Vaccines: a Systematic Review and Appraisal of the Potential to Exploit Biodefense Vaccines for Public Health Purposes. PLoS Negl Trop Dis. 2012;6(1):e1488 10.1371/journal.pntd.0001488 22303489 PMC3269417

[6] Chua K, See K, Thong K, Puthucheary S. DNA Fingerprinting of Human Isolates of Burkholderia Pseudomallei from Different Geographical Regions of Malaysia. Trop Biomed. 2010;27(3):517–24. 21399594

[7] Hassan MRA, Pani SP, Peng NP, Voralu K, Vijayalakshmi N, Mehanderkar R, et al Incidence, Risk Factors and Clinical Epidemiology of Melioidosis: A Complex Socio-Ecological Emerging Infectious Disease in the Alor Setar Region of Kedah, Malaysia. BMC Infect Dis. 2010;10:302–. 10.1186/1471-2334-10-302 PMC2975659. 20964837 PMC2975659

[8] How S, Ng K, Jamalludin A, Shah A, Rathor Y. Melioidosis in Pahang, Malaysia. Med J Malaysia. 2005;60(5):606–13. 16515112

[9] Pagalavan L. Melioidosis: the Johor Bahru Experience. Med J Malaysia. 2005;60(5):599 16515111

[10] Puthucheary S, Parasakthi N, Lee M. Septicaemic Melioidosis: a Review of 50 Cases from Malaysia. Trans R Soc Trop Med Hyg. 1992;86(6):683–5. 1287945 10.1016/0035-9203(92)90191-e

[11] Nathan S, Chieng S, Kingsley PV, Mohan A, Podin Y, Ooi M-H, et al Melioidosis in Malaysia: Incidence, Clinical Challenges, and Advances in Understanding Pathogenesis. Trop Med Infect Dis. 2018;3(1):25. 10.3390/tropicalmed3010025 PMC6136604 30274422

[12] Whitaker HJ, Paddy Farrington C, Spiessens B, Musonda P. Tutorial in Biostatistics: The Self Controlled Case Series Method. Stat Med. 2006;25(10):1768–97. 10.1002/sim.2302 16220518

[13] Whitaker HJ, Hocine MN, Farrington CP. The Methodology of Self-Controlled Case Series Studies. Stat Methods Med Res. 2009;18(1):7–26. 10.1177/0962280208092342 18562396

[14] Limmathurotsakul D, Kanoksil M, Wuthiekanun V, Kitphati R, Day NP, Peacock SJ. Activities of Daily Living Associated With Acquisition of Melioidosis in Northeast Thailand: a Matched Case-Control Study. PLoS Negl Trop Dis. 2013;7(2):e2072 10.1371/journal.pntd.0002072 23437412 PMC3578767

[15] Corkeron ML, Norton R, Nelson PN. Spatial Analysis of Melioidosis Distribution in a Suburban Area. Epidemiol Infect. 2010;138(9):1346–52. Epub 2010/01/22. 10.1017/S0950268809991634 20092666

[16] SPSS I. IBM SPSS Statistics for Windows, Version 20.0 New York: IBM Corp 2011.

[17] Team RC. R: A Language and Environment for Statistical Computing. 2013.

[18] Desktop EA. Release 10 Redlands, CA: Environmental Systems Research Institute 2011;437:438.

[19] Kulldorff M. SaTScan-Software for the Spatial, Temporal, and Space-Time Scan Statistics. Boston: Harvard Medical School and Harvard Pilgrim Health Care 2010.

[20] Kulldorff M. A Spatial Scan Statistic. Commun Stat Theory Methods. 1997;26(6):1481–96.

[21] Nachtergaele F. Mapping Land Use Systems at Global and Regional Scales for Land Degradation Assessment Analysis. 2010.

[22] Center for International Earth Science Information Network—CIESIN—Columbia University, United Nations Food + Agriculture Programme—FAO, Centro Internacional de Agricultura Tropical—CIAT. Gridded Population of the World, Version 3 (GPWv3): Population Count Grid Palisades, NY: NASA Socioeconomic Data and Applications Center (SEDAC); 2005.

[23] Maier MJ. DirichletReg: Dirichlet Regression for Compositional Data in R. 2014.

[24] Raja N, Ahmed M, Singh N. Melioidosis: an Emerging Infectious Disease. J Postgrad Med. 2005;51(2):140 16006713

[25] Malaysia DoS. Population Distribution and Basic Demographic Characteristic Report 2010 2011.

[26] Limmathurotsakul D, Wongratanacheewin S, Teerawattanasook N, Wongsuvan G, Chaisuksant S, Chetchotisakd P, et al Increasing Incidence of Human Melioidosis in Northeast Thailand. Am J Trop Med Hyg. 2010;82(6):1113–7. 10.4269/ajtmh.2010.10-0038 20519609 PMC2877420

[27] Merianos A, Patel M, Lane J, Noonan C, Sharrock D, Mock P, et al The 1990–1991 Outbreak of Melioidosis in the Northern Territory of Australia: Epidemiology and Environmental Studies. Southeast Asian J Trop Med Public Health. 1993;24(3):425–35. 7512752

[28] Suputtamongkol Y, Chaowagul W, Chetchotisakd P, Lertpatanasuwun N, Intaranongpai S, Ruchutrakool T, et al Risk Factors for Melioidosis and Bacteremic Melioidosis. Clin Infect Dis. 1999;29(2):408–13. 10.1086/520223 10476750

[29] Currie BJ, Jacups SP, Cheng AC, Fisher DA, Anstey NM, Huffam SE, et al Melioidosis Epidemiology and Risk Factors from a Prospective Whole-Population Study in Northern Australia. Trop Med Int Health. 2004;9(11):1167–74. 10.1111/j.1365-3156.2004.01328.x 15548312

[30] Kaestli M, Mayo M, Harrington G, Ward L, Watt F, Hill JV, et al Landscape Changes Influence the Occurrence of the Melioidosis Bacterium Burkholderia Pseudomallei in Soil in Northern Australia. PLoS Negl Trop Dis. 2009;3(1):e364 10.1371/journal.pntd.0000364 19156200 PMC2617783

[31] Wuthiekanun V, Smith MD, Dance DA, White NJ. Isolation of Pseudomonas Pseudomallei from Soil in North-Eastern Thailand. Trans R Soc Trop Med Hyg. 1995;89(1):41–3. 7538233 10.1016/0035-9203(95)90651-7

[32] Singapore MoH. Melioidosis in Singapore. Epidemiological News Bulletin 1995.

[33] Hussein Z, Taher SW, Gilcharan Singh HK, Chee Siew Swee W. Diabetes Care in Malaysia: Problems, New Models, and Solutions. Ann Glob Health. 2015;81(6):851–62. Epub 2016/04/25. 10.1016/j.aogh.2015.12.016 . 27108152

[34] Jan Mohamed HJ, Yap RW, Loy SL, Norris SA, Biesma R, Aagaard-Hansen J. Prevalence and determinants of overweight, obesity, and type 2 diabetes mellitus in adults in Malaysia. Asia Pac J Public Health. 2015;27(2):123–35. 10.1177/1010539514562447 25524952

[35] Cheng AC, Limmathurotsakul D, Chierakul W, Getchalarat N, Wuthiekanun V, Stephens DP, et al A Randomized Controlled Trial of Granulocyte Colony-Stimulating Factor for the Treatment of Severe Sepsis Due to Melioidosis in Thailand. Clin Infect Dis. 2007;45(3):308–14. 10.1086/519261 17599307

[36] Bagdade JD, Root RK, Bulger RJ. Impaired Leukocyte Function in Patients with Poorly Controlled Diabetes. Diabetes. 1974;23(1):9–15. 4809622 10.2337/diab.23.1.9

[37] Goetz MB, Proctor RA. Normalization of Intracellular Calcium: A Sweet Solution to Neutrophil Dysfunction in Diabetes? Ann Intern Med. 1995;123(12):952–4. 7486493 10.7326/0003-4819-123-12-199512150-00011

[38] Stephens D, Fisher D, Currie B. An Audit of the Use of Granulocyte Colony Stimulating Factor in Septic Shock. Intern Med J. 2002;32(4):143–8. 11951925 10.1046/j.1445-5994.2002.00195.x

[39] Patz JA, Olson SH, Uejio CK, Gibbs HK. Disease Emergence from Global Climate and Land Use Change. Med Clin North Am. 2008;92(6):1473–91. 10.1016/j.mcna.2008.07.007 19061763

[40] Wilcox BA, Ellis B. Forests and Emerging Infectious Diseases of Humans. UNASYLVA-FAO-. 2006;57(2):11.

